# pH and Phosphate Induced Shifts in Carbon Flow and Microbial Community during Thermophilic Anaerobic Digestion

**DOI:** 10.3390/microorganisms8020286

**Published:** 2020-02-20

**Authors:** Nina Lackner, Andreas O. Wagner, Rudolf Markt, Paul Illmer

**Affiliations:** Department of Microbiology, Universität Innsbruck, 6020 Innsbruck, Austria; andreas.wagner@uibk.ac.at (A.O.W.); rudolf.markt@uibk.ac.at (R.M.); paul.illmer@uibk.ac.at (P.I.)

**Keywords:** pH, phosphate, microbial community, carbon flow, next-generation sequencing, biogas, buffer, volatile fatty acids

## Abstract

pH is a central environmental factor influencing CH_4_ production from organic substrates, as every member of the complex microbial community has specific pH requirements. Here, we show how varying pH conditions (5.0–8.5, phosphate buffered) and the application of a phosphate buffer per se induce shifts in the microbial community composition and the carbon flow during nine weeks of thermophilic batch digestion. Beside monitoring the methane production as well as volatile fatty acid concentrations, amplicon sequencing of the 16S rRNA gene was conducted. The presence of 100 mM phosphate resulted in reduced CH_4_ production during the initial phase of the incubation, which was characterized by a shift in the dominant methanogenic genera from a mixed *Methanosarcina* and *Methanoculleus* to a pure *Methanoculleus* system. In buffered samples, acetate strongly accumulated in the beginning of the batch digestion and subsequently served as a substrate for methanogens. Methanogenesis was permanently inhibited at pH values ≤5.5, with the maximum CH_4_ production occurring at pH 7.5. Adaptations of the microbial community to the pH variations included shifts in the archaeal and bacterial composition, as less competitive organisms with a broad pH range were able to occupy metabolic niches at unfavorable pH conditions.

## 1. Introduction

The application of anaerobic digestion to produce energy from biogas is a well-established technique to treat organic waste and has been used since the late 19^th^ century [1,2]. The anaerobic degradation is generally divided into four consecutive steps performed by different physiological groups of microorganisms [3]. After initial hydrolysis and the subsequent fermentation steps (acidogenesis and acetogenesis), methanogenic substrates, mainly acetate, H_2_, and CO_2_, are consumed to form biogas, mainly consisting of CH_4_ and CO_2_. The degradation of acetate may follow two different pathways depending on the dominant methanogen. Acetoclastic methanogenic archaea (AMA) directly metabolize acetate to CH_4_ and CO_2_, while hydrogenotrophic methanogenic archaea (HMA) cooperate with syntrophic acetate oxidizing bacteria (SAOB) to degrade acetate to CH_4_. The composition and efficiency of a microbial community in an anaerobic methanogenic system is affected by a range of environmental factors, e.g., substrate composition, microbial composition of the inoculum, temperature, and pH [4,5,6,7,8]. 

Investigating anaerobic digestion in batch systems requires stable pH conditions and therefore the application of a buffer. Phosphate buffers (P-buffers) are widely used to cultivate anaerobic microorganisms, including AMA and HMA, as they span a wide pH range [9,10]. The phosphate concentration suitable for bacterial growth is highly variable, ranging from 0.3–300 mM [11]. Too high concentrations of phosphate may inhibit growth as well as secondary metabolism in bacteria [11]. In this context, possible modes of action of phosphate inhibition include changes of extracellular pH, interference with trace metal metabolism and inhibition of the alkaline phosphatase [11]. In anaerobic digestion, the addition of phosphate might have positive as well as negative effects, depending on the added amount and the chemical and microbial status of the system. In small doses, phosphate addition may encounter phosphorous deficiencies, suppress ammonia inhibition, or precipitate ammonia together with magnesium to form struvite [12,13]. In contrast, high doses of phosphate may have an adverse effect on CH_4_ production, with the inhibitory level ranging from 50 to >100 mM and AMA being especially sensitive [14].

pH affects all aspects of microbial life including enzyme activity, protein stability, structure of nucleic acids, transmembrane electrical potential, and membrane permeability of small organic molecules, e.g., ammonia and volatile fatty acids (VFA) [15]. Although microorganisms possess a range of mechanisms to control and stabilize their intracellular pH, this homeostatic process is limited. Mechanisms and capacity of homeostasis vary between different groups of organisms and include cytoplasmic buffering, adaptations in the cell membrane structure, active ion transport, and shifts in fermentation products [15]. The capacity for pH homeostasis determines the pH range in which growth can occur, defining microorganisms as acidophilic (pH optima 0.5–5), neutrophilic (pH optima 5–9) or alkaliphilic (pH optima 9–12) [15]. 

In anaerobic digestion, the pH preferences of the engaged microorganisms are diverse, as fermentative organisms have lower pH optima than methanogens [16]. Therefore, anaerobic digestion for biogas production is sometimes divided in a two-stage or three-stage process to enable optimal pH conditions for every microbial group [17]. If acidogenesis and methanogenesis were not spatially separated, the optimal pH for successful biogas production was generally found to be 6.8–7.4 [2,16]. Several types of process disturbances, caused by for example overload conditions, may; however, lead to the accumulation of acids and a concomitant drop in pH [1,18]. Besides the microbial community and CH_4_ yield, the prevailing pH affects the amount and the composition of the produced VFA as well as the protonation and therefore toxicity of VFA and ammonia [5,16,19,20,21,22]. Previous publications investigating the effect of experimentally changed pH conditions during anaerobic digestions are not frequent and mostly concern mesophilic reactors. 

O’Flaherty et al. (1998) [23] investigated maximal growth rates of methanogens, sulfate reducing bacteria, and SAOB in pure cultures and/or anaerobic sludges in dependence of the pH. They observed optima for single strains or trophic groups between pH 6.8 and 8.5, with broader optimal pH ranges in sludge samples than pure cultures [23]. Zhai et al. (2015) [20] found maximal CH_4_ yields at an initial pH of 7.5 and a process failure at an initial pH of 6 (neither degradation of VFA nor CH_4_ production) during mesophilic digestion of kitchen waste and cow manure. The mesophilic digestion of pig manure at pH 6–8 led to a maximum CH_4_ production at pH 7 and a shift in the dominant methanogenic genus at pH 6 and 8 [24]. These results were in accordance with Zang et al. (2009) [21], who found the highest CH_4_ yields at pH 7 under thermophilic conditions, when digesting waste activated sludge. Hao et al. (2012) [19] used acetate as substrate for thermophilic digestion and found that methanogenesis was totally inhibited at pH 5, while it was mainly hydrogenotrophic or acetoclastic at pH 5.5 or 6–6.5, respectively. Data on the microbial community composition at different pH regimes can only be found in a study by Boaro et al. (2014) [25]. They performed an integrated omics analysis of a mesophilic cellulose degrading community, which was disturbed by a onetime acid pulse. There, it could be shown that the pH perturbation led to a decrease in cellulolytic activity and surprisingly an increase in alpha diversity, but the dominant members of the community persisted [25].

The object of the present investigation is to analyze the effect of varying pH conditions on all steps of the thermophilic anaerobic digestion process. To achieve stable pH conditions in batch reactors, the application of a buffer was inevitable. Therefore, the effect of the applied P-buffer on the system is separately analyzed and discussed. The present study aims to (i) assess the impact of the applied P-buffer on the archaeal and bacterial community, including possible pH dependent phosphate effects, (ii) describe shifts in the microbial community due to pH alterations and identify microorganisms that are more tolerant against atypical pH conditions, and (iii) bring shifts in the microbial community and the physiological abilities of their members in context with shifts in the carbon pools during the multi-step anaerobic digestion process. To our knowledge, this is the first study to compare biochemical gas and VFA data with next-generation sequencing (NGS) community profiles in a pH or phosphate influenced thermophilic system.

## 2. Materials and Methods

### 2.1. Experimental Setup

Serum flasks with a total volume of 250 mL were filled with 50 mL sodium carboxymethyl cellulose solution (10 g/L, Roth, Graz, Austria) and 20 mL phosphate buffer (P-buffer). The application of carboxymethyl cellulose as a substrate was necessary to ensure a growing microbial community, in which compositional changes would occur in a suitable time span. The P-buffer contained varying amounts of 0.5 M Na_2_HPO_4_ and 0.5 M KH_2_PO_4_ to obtain the intended pH variations from pH 5.0 to 8.5 (final phosphate concentration in the serum flasks 0.1 M). Subsequently, the headspace of the flasks was flushed with N_2_ gas to guarantee anoxic conditions. Then, 1:5 diluted fermenter sludge (DFS) from a thermophilic plug flow fermenter in Roppen (Tirol) was used as an inoculum. For technical details and process parameters of the fermenter, please refer to Illmer and Gstraunthaler (2009) [26]. Prior to the addition of 30 mL, the pH of the DFS was adjusted with 1 M HCl and 0.2 M NaOH for the acidic and alkaline variants, respectively (volumetric differences were compensated with oxygen-free distilled water). An additional unbuffered variant contained distilled water instead of the P-buffer and DFS with an uncontrolled pH of 7.8. All samples were incubated for 9 weeks at 50 °C with measurement time points on day 0, 3, 7, 14, 21, 28, 42, and 63. As it became obvious that the P-buffer per se had an impact on methanogenesis, a supplementary experiment was conducted to assess this effect at different phosphate concentrations (0, 12.5, 25, 50, and 100 mM). It was setup and incubated as described for the main experiment, but its evaluation was restricted to gas analyses.

### 2.2. Physio-Chemical Analyses

The volume of the produced gas was calculated based on the overpressure in the flasks (digital precision manometer GDH 200-13, Greisinger electronic, Regenstauf, Germany) and the ambient pressure (data from Zentralanstalt für Meteorologie und Geodynamik, Austria) according to [27]. The gas composition was measured with a Shimadzu GC2010 gas chromatograph (Shimadzu, Kyōto, Japan) equipped with a flame ionization detector (CH_4_) and a thermal conductivity detector (H_2_, CO_2_), as described in [28]. VFA were analyzed in undiluted, sterile-filtrated liquid samples via high performance liquid chromatography as recommended in [29]. The total amount of dissolved ammonia (NH_3_ and NH_4_^+^) concentration in the inoculum was determined in three technical parallels via flow injection analysis on a Prominence HPLC system (Shimadzu, Kyōto, Japan) as described in [30]. The pH value was measured in undiluted samples immediately after sampling with a pH 340i/SET electrode (WTW, Weilheim, Germany).

### 2.3. 16S RNA Gene Library Preparation and Amplicon Sequencing

For amplicon sequencing, the V4 region of the 16S gene was targeted using the primer pair 515f/806r [31,32]. Library preparation comprised two subsequent PCR runs. During PCR 1 the adapter-sequence was attached to the target region (30 cycles). These adapters were needed in the second PCR to attach sample specific barcodes (5 cycles). For DNA extraction, 1 mL of culture fluid of each serum flask was centrifuged at 20,000× g for 10 min. Subsequently, 0.8 mL of supernatant was removed and the pellet was resuspended in the remaining fluid. DNA extraction was performed with a NucleoSpin^®^ Soil kit (Macherey-Nagel, Düren, Germany) as described in the manufacturer’s protocol. DNA was quantified using a Quant-iT™ PicoGreen™ dsDNA Assay Kit (Invitrogen, Waltham, Massachusetts, USA) and a multimode flourometer Zenyth 3100 (Anthos, Salzburg, Austria). Subsequently, the DNA was diluted to 0.5 ng/µL with PCR-grade distilled water. PCR 1 contained, per 25 µL reaction volume, 12.5 µL NEBNext^®^ Ultra™ II Q5^®^ Master Mix (New England Biolabs, Ipswich, Massachusetts, USA), 1.25 µL of each primer (final concentration 0.5 µM) and 10 µL diluted DNA extract. After the initial heat activation of the proof-read polymerase for 30 s at 98 °C, each cycle comprised 10 s at 98 °C, 30 s at 58 °C, and 20 s at 72 °C. The final elongation step was 2 min at 72 °C. PCR 2 contained, per 20 µL reaction volume, 10 µL NEBNext Ultra II Q5 Master Mix, 1+1 µL of the respective barcode primer pair (final concentration 0.5 µM), 3 µL PCR grade water, and 5 µL of 1:5 diluted PCR product. The success of both PCR steps was controlled by performing agarose gel electrophoresis (1.5% agarose, 15 min, 100 mV). Subsequently, a library was prepared with equal amounts of DNA per sample with a concentration of 47 ng/µL and cleaned up using a Hi Yield^®^ Gel/PCR DNA fragment extraction kit (Süd-Laborbedarf GmbH, Gauting, Germany). Finally, 100 µL of the library were sent to Microsynth AG (Balgach, Switzerland) for amplicon sequencing (Illumina MiSeq 2x 250 bp paired end read). All steps described above were also performed with a mock community (ZymoBIOMICS Microbial Community Standard, Zymo Research, Irvine, California, USA) to validate the library construction.

### 2.4. Data Analysis and Statistics

Raw reads from amplicon sequencing were processed and analyzed with mothur v.1.39.5 [33]. The applied pipeline was optimized using the mock community, leading to a root-mean-square deviation of 8.15% from the predicted mock community composition. The sequences were screened for deviating read lengths, ambiguities and sequences containing more than 7 homopolymers. The remaining sequences were aligned to the SILVA database release 132 ribosomal SSU (release date 12.12.17) [34,35]. Chimeras were removed using the VSEARCH 2.3.4 algorithm [36] and sequences classified using the k-Nearest Neighbor algorithm. Quality-filtered reads were submitted to the NCBI GenBank under the BioProject number PRJNA559798. The sequence counts were subsampled to the smallest sample size of 16,726 reads/sample. Operational taxonomic units (OTUs) in the results and discussions sections summarize sequences with the same taxonomic classification on genus level. OTUs based on 97% similarity led to identical results and were therefore not incorporated in the present publication. OTUs on genus level contributing not more than 0.1% of total reads in at least one sample, or being present in less than 3 samples, were summarized as rare OTU before further statistical analyses. The resulting OTU table was analyzed concerning the alpha diversity of the microbial communities using the Shannon index as implemented in mothur. Significant differences (*p* < 0.01) in the Shannon index were identified using one-way ANOVA or Welch-ANOVA depending on the homogeneity of variance in IBM SPSS Statistics 25 (International Business Machines Corporation, Armonk, New York, USA), after testing for normality with the Levene statistic. Further, cluster analyses of the box-cox transformed abundance data were performed in PAST 3 [37] (Bray-Curtis distances, UPGMA). Indicator genera for different subclusters were identified by calculating group size-equalized indicator values (IndVal) with 1000 permutations as implemented in mothur [38]. The thresholds for relevant indicator genera were set to *p* < 0.01 and IndVal > 50. Clusters and indicator genera were visualized with Dendroscope (version 3.6.3, built 1 Aug 2019) [39] and CorelDRAW^®^ Graphics Suite X7 (Corel Corporation, Ottawa, Ontario, Canada). Significant differences (*p* < 0.01) in the microbial communities at phylum level were calculated using one-way or two-way PERMANOVA in PAST 3 (box-cox transformed data, Bray-Curtis distances, 9999 permutations). The compositions of the microbial communities at order and phylum level were charted using Microsoft Excel 2010 (Microsoft Corporation, Redmond, Washington, USA).

Data on carbon pools were tested for normality and homogeneity of variance with the Shapiro–Wilk and Levene statistic, respectively. Due to variables lacking homogeneity of variance, the robust Welch-ANOVA was used to analyze significant differences between different variants at single incubation time points (IBM SPSS Statistics 25). The alpha level for significant differences was 0.01. Graphs were created in Statistica 12 (StatSoft^®^, Tulsa, Oklahoma, USA) and show mean ± standard deviations of triplicates. All biochemical data are given per L reactor fluid. The amount of free dissolved ammonia (NH_3_) at different pH values was calculated based on the pKa for NH_3_ at 50 °C (=8.54) and the measured amount of total ammonia using the Henderson–Hasselbalch equation. 

## 3. Results

### 3.1. Starting Conditions and Microbial Community of the Inoculum

At the beginning of the experiments, the pH variants showed minor but significant differences in CH_4_, CO_2_, acetate, and propionate concentrations, probably due to chemical pH effects such as altered solubility of gases and volatility of VFA (Table S1). In contrast, P-buffered pH 7.5 samples did not differ significantly from the unbuffered control samples regarding these chemical parameters. The total ammonia concentration in the 1:5 diluted fermenter sludge was 71.2 ± 1.4 mmol/L, meaning that the final concentration in the serum flasks was in the range of 21.4 ± 0.4 mmol/L. Therefore, the calculated concentration of free dissolved ammonia (NH_3_) varied between 0.0 mmol/L at pH 5.0, 1.8 mmol/L at pH 7.5, and 10.2 mmol/L at pH 8.5 on day 0. 

**Figure 1:**
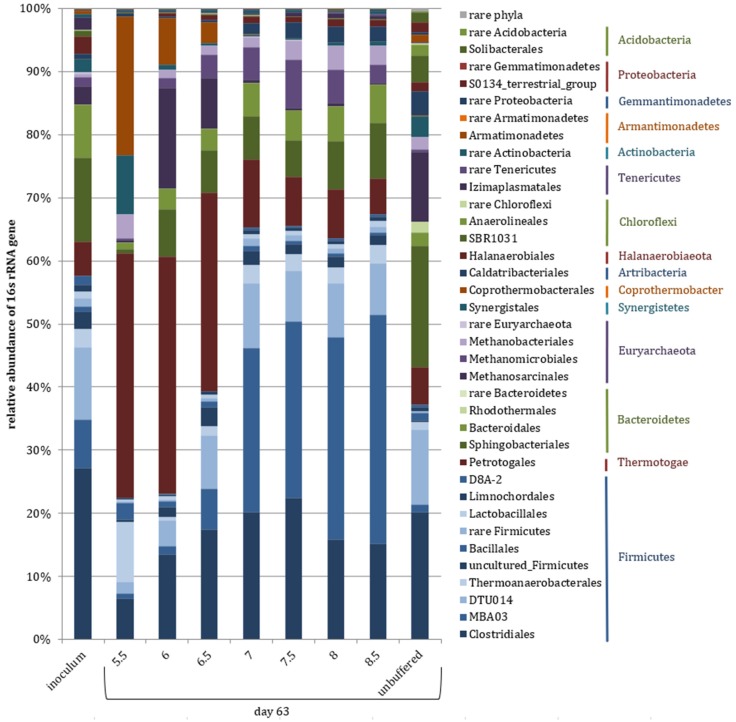
Microbial community composition at phylum and order level of the inoculum as well as samples incubated for 63 days at varying pH conditions (100 mM P-buffered) or unbuffered. Phyla or orders with not more than 1% abundance in at least one sample were summarized as rare phyla or rare members of the particular phylum, respectively (means of triplicates).

**Figure 2:**
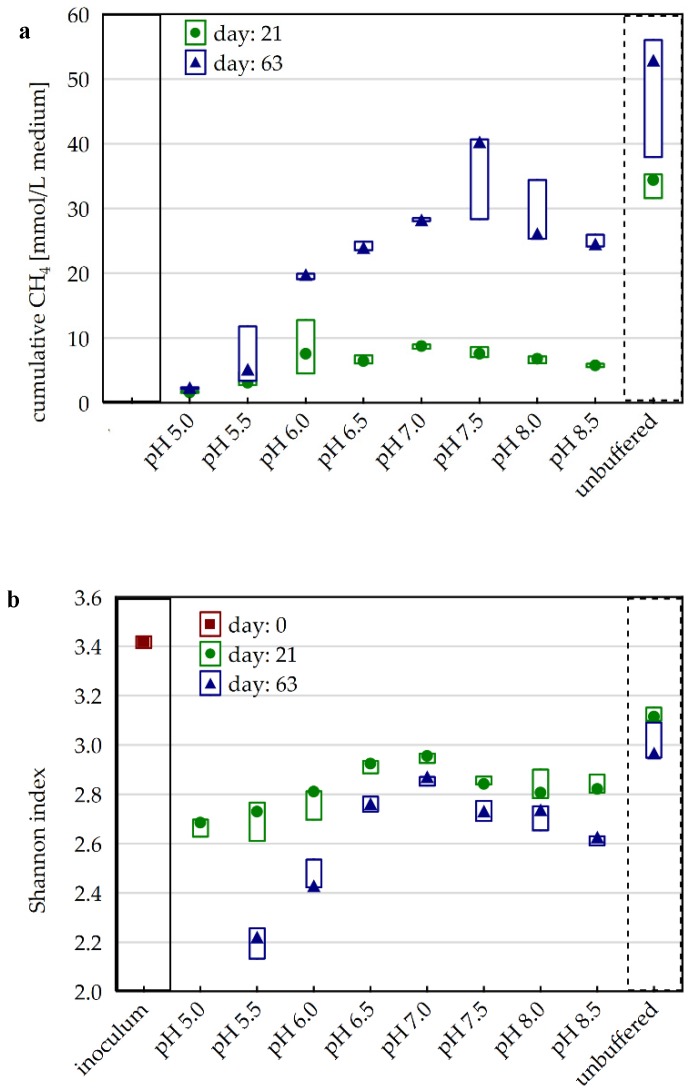
(**a**) Cumulative CH_4_ production after 21 and 63 days of incubation under various pH conditions including an unbuffered control (minimum, median, and maximum of triplicates); (**b**) Diversity of the microbial community of the inoculum and after 21 and 63 days of incubation under various pH conditions including an unbuffered control (minimum, median, and maximum of triplicates). Calculated based on 16S amplicon sequencing data. Samples with pH 5 of day 63 are missing due to too low DNA concentrations.

The microbiome of the inoculum exhibited the highest alpha diversity of all samples and consisted of 56% Firmicutes, 22% Bacteroidetes, 6% Thermotogae, 6% Euryarchaeota and less abundant Halanaerobiaeota, Synergistes, Tenericutes, and Chloroflexi (Figure 1 and Figure 2b). The methanogenic community was dominated by the genera *Methanosarcina* (4%) and *Methanoculleus* (1%), meaning that they were almost equally abundant considering that the mean 16S copy numbers of the two genera are three and one, respectively.

### 3.2. Impact of P-Buffer on the Carbon Flow

**Figure 3:**
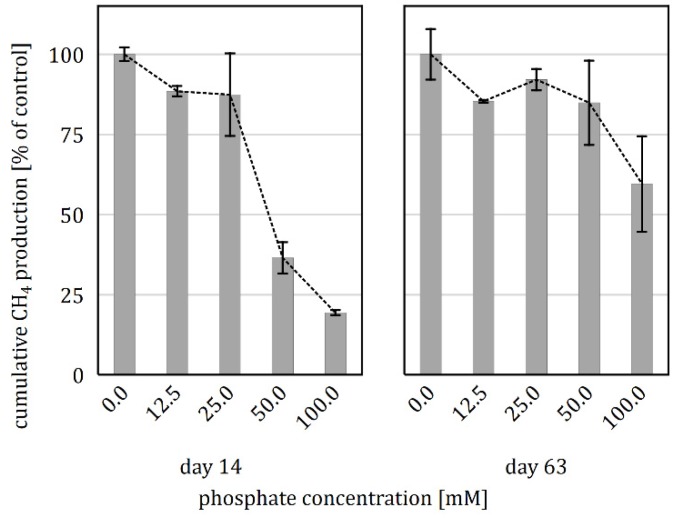
Effect of P-buffer addition in various concentrations on the cumulative CH_4_ production after 14 and 63 days of anaerobic incubation relative to the production of the unbuffered control (means ± standard deviations of triplicates).

Parallel to the pH variants, an unbuffered variant was prepared, which showed a stable pH of 7.7 ± 0.2 throughout the entire incubation. The comparison of the unbuffered with the P-buffered pH 7.5 variant showed that cumulative CH_4_ production was inhibited in the presence of P-buffer, with significant differences during the first four weeks of incubation (Figure 2a). Therefore, a supplementary experiment to test varying P-buffer concentrations was conducted. The results showed that compared with the 0.0 mM variant, the amendment of 12.5, 25.0, 50.0, and 100.0 mM P-buffer led to an increasing reduction in cumulative CH_4_ yield, with relative differences decreasing over time (Figure 3).

**Figure 4:**
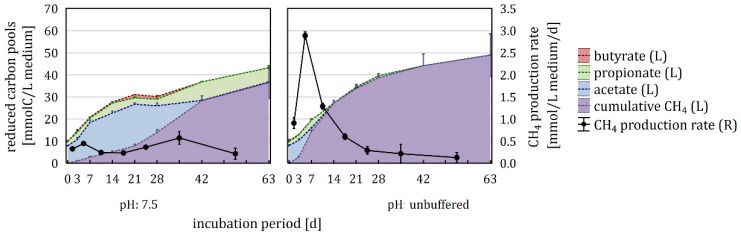
Reduced carbon (butyrate, propionate, acetate, and cumulative CH_4_ (left axis)) and CH_4_ production rate (right axis) during the anaerobic incubation of samples buffered at pH 7.5 with 100 mM P-buffer and unbuffered samples (pH 7.7) (means ± standard deviations of triplicates).

To further assess this phenomenon, the unbuffered samples of the main experiment were included in the VFA analysis and compared with the P-buffered pH 7.5 samples, as they exhibited very similar pH conditions. The sum of reduced carbon (sum of carbon from butyrate, propionate, acetate and cumulative CH_4_) was calculated as an indicator for the rate of hydrolysis and acidogenesis. Until day 14, the increase in reduced carbon was almost identical, but started to differentiate thereafter, resulting in a significantly lower amount of reduced carbon in P-buffered samples on day 28 (Figure 4, Table S1). Further, the composition of the reduced carbon pools varied distinctly between the unbuffered and buffered samples, with buffered samples exhibiting significantly lower cumulative CH_4_ amount from day 3 to 28. In this context, propionate and acetate differed significantly between buffered and unbuffered samples from day 21 to 28 and day 7 to 28, respectively. In unbuffered samples, acetate, propionate, and butyrate were depleted until day 14, 14, and 7, respectively. In contrast, acetate and butyrate concentrations increased during the initial weeks of incubation before they decreased until their exhaustion on day 42 and propionate persisted until the end of the incubation in buffered samples (Figure 4). Maximal CH_4_ production rates of almost 3 mmol/L medium/d were reached between day 3 and 7 in unbuffered samples, while buffered samples showed two maxima between day 3 and 7 as well as between day 28 and 42, that were both in the range of 0.5 mmol/L medium/d. 

### 3.3. Effect of pH on Carbon Flow

**Figure 5:**
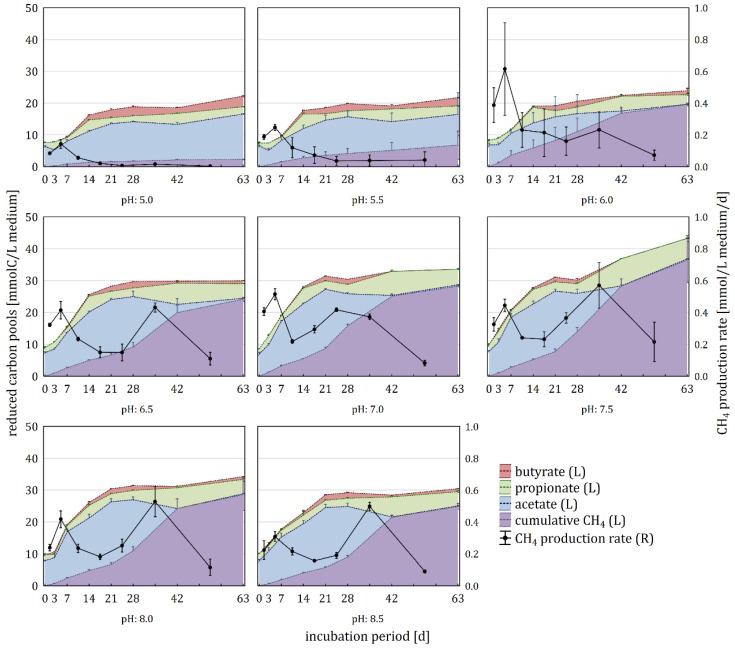
Reduced carbon (butyrate, propionate, acetate, and cumulative CH_4_ (left axis)) and CH_4_ production rate (right axis) during the anaerobic incubation at varying pH values (means ± standard deviations of triplicates).

Using 100 mM P-buffer, pH variations from pH 5.0 to 8.5 were prepared. The pH of the variants stayed adequately constant during the incubation period of 63 days with a mean and maximal absolute deviation from the intended pH of 0.25 and 0.39 pH units, respectively. In the first three weeks, the amount of reduced carbon increased up to approximately 20 and 30 mmol/L medium in samples with pH 5.0–6.0 and 6.5–8.5, respectively (Figure 5 and Figure S3). In the subsequent six weeks, distinct increases in reduced carbon could only be observed in pH 7.5 variants. 

On day 21 and 63, all carbon pools differed significantly depending on the applied pH (Table S1). Butyrate could not be detected at the start of the experiment but accumulated in all variants during the first three to four weeks. On day 21, the highest concentrations were found in pH 5.0 samples. Subsequently, butyrate levels decreased until day 42, followed by a second increase in all samples except pH 7.0 and 7.5, in which butyrate was completely exhausted. Propionate concentrations fluctuated during the nine weeks of incubation, with peaks on day 3, 14, and 42 and highest concentrations reached at neutral pH. Acetate increased until day 28 and 21 in samples with pH 5.0–6.0 and pH 6.5–8.5, respectively. In variants with a pH between 6.5 and 8.5, the accumulated acetate reached its highest levels and was depleted until day 42 (pH 7.0–8.5) or day 63 (pH 6.0–6.5), while it persisted in the more acidic samples. 

CO_2_ in the headspace increased steadily in all samples during the incubation period, reaching its maximum at pH 6.5 on day 63 (Table S1), with headspace concentrations being also influenced by the pH dependent solubility. CH_4_ production occurred in two phases, from day 0 to 21 and from day 21 to 63. In variants with a pH ≥ 6.0, CH_4_ production occurred in both phases, leading to final CH_4_ yields between 19.60 and 36.43 mmol/L medium. In more acidic samples, methanogenesis occurred mainly in the first phase, leading to final CH_4_ yields between 2.28 and 6.79 mmol/L medium. The highest cumulative CH_4_ production until day 21 and 63 was found at pH 7.0 and 7.5, respectively (Figure 2a). Apart from the carbon flow, the H_2_ production was monitored, with the highest amounts being reached on day 7 in pH 5.0 samples (0.20 ± 0.09 mmol/L). On day 21, neutral samples showed the lowest cumulative H_2_ amount and on day 63 H_2_ levels were near zero in all samples.

### 3.4. Impact of the P-Buffer on the Microbial Community

**Figure 6:**
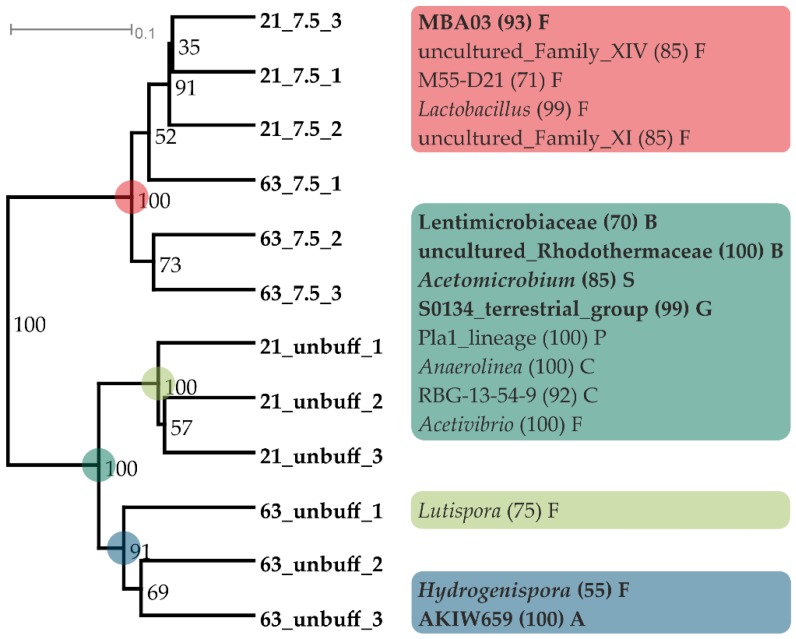
Shifts in the microbial community due to the addition of 100 mM P-buffer (pH 7.5) compared with unbuffered samples (pH 7.7). Cluster analysis of box-cox transformed abundance data on day 21 and 63 with bootstrap numbers at nodes (Bray-Curtis, UPGMA). Colored dots link distinct subclusters with their respective indicator genera. Numbers in brackets state the IndVal (*p* < 0.01) and genera with a maximum abundance above 1% are written in bold letters. Sample abbreviations state the sampling day and if they were buffered at pH 7.5 or unbuffered. Single letters state the phyla the indicator genera; F: Firmicutes, B: Bacteriodetes, S: Synergistes, G: Gemmatimonadetes, P: Planctomyces, C: Chloroflexi, A: Acidobacteria.

As for the carbon flow, the pH 7.5 buffered and the unbuffered samples (stable pH of 7.7 ± 0.2) were further used to assess the effect of the P-buffer addition per se on the microbial community. The data revealed that the alpha diversity (Shannon index) on genus level was significantly reduced in buffered samples compared with unbuffered samples on day 21 and 63 (one-way ANOVA) (Figure 2b). Further, the community composition at phylum level differed significantly between buffered and unbuffered samples, but not significantly with time (two-way PERMANOVA), with higher relative abundances of Firmicutes and Thermotogae, and lower relative abundances of Bacteroidetes, Synergistes, Atribacteria, and Chloroflexi in P-buffered samples (Figure 1). The community composition at genus level was evaluated for unbuffered and buffered samples from day 21 and 63 via a cluster analysis with subsequent calculation of indicator genera for distinct clusters. The analysis separated the samples in two main clusters comprising either buffered or unbuffered samples, while the incubation duration was less decisive (Figure 6). Indicator genera for unbuffered samples with more than 1% maximal abundance were an unclassified genus of Lentimicrobiaceae, uncultured Rhodothermaceae, *Acetomicrobium*, S0134_terrestrial_group, *Hydrogenispora* (only day 63), and AKIW659 (only day 63), while the cluster with buffered samples exhibited only one abundant indicator genus, an uncultured Firmicutes group named MBA03. 

**Figure 7:**
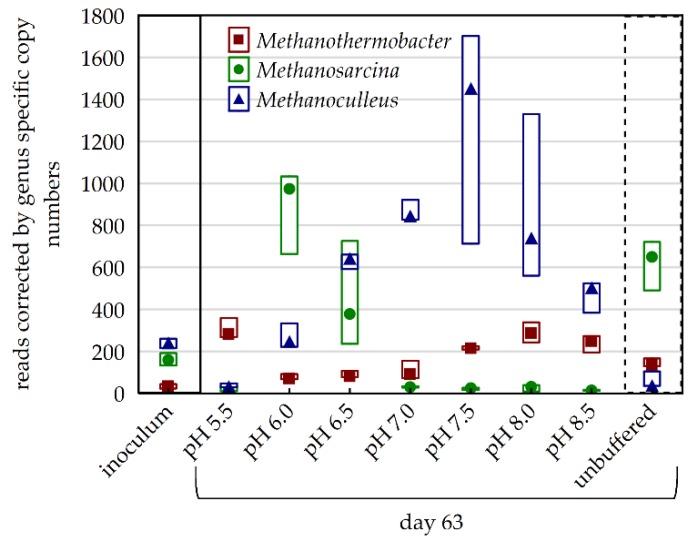
16S copy number corrected reads of methanogenic genera of the inoculum and after 63 days of incubation at varied pH conditions including an unbuffered control (minimum, median, and maximum of triplicates).

While the relative abundance of Euryarchaeota was almost identical in buffered and unbuffered samples on day 63, the composition of the methanogenic community at genus level varied strongly. The NGS reads used to compare the relative abundances of the methanogenic genera were corrected by the mean 16S copy numbers of the genera (rrnDB version 5.5, 20.09.18) [40] to allow the direct comparison between them. On day 63 the unbuffered samples were dominated by *Methanosarcina* species, while *Methanoculleus* was the most abundant methanogenic genus in P-buffered samples (Figure 7). 

### 3.5. Effect of pH on the Microbial Community

The microbial communities of the pH-controlled samples changed distinctively during the incubation period of nine weeks in all variants, leading to pH-specific compositions. Samples with a pH of 5.0 did not contain any detectable DNA after extraction on day 63 and are therefore missing in the NGS analysis. A continuous adaptation of the microbial communities to the respective pH conditions could be observed from day 0 to 63 (Figure 1, Figure S2). In this context, the alpha diversity decreased over time in all samples, with the weakest decrease in neutral pH variants (Figure 2b). The Shannon index (Welch-ANOVA) and the microbial communities at phylum and genus level (one-way PERMANOVA) varied significantly between the pH values on day 21 as well as day 63. To avoid repetitions, the microbial compositions on day 63 are described in more detail, whereas the results for day 21 are shown in Figures S1 and S2. As it was not possible to find reliable data on 16S copy numbers for all OTUs, the relative abundances in this manuscript relate to 16S reads, if not stated otherwise.

On day 63, bacterial phyla, which showed mean relative abundances above 10% in at least one pH variant comprised Firmicutes, Thermotogae, Bacteriodetes, Coprothermobacteraeota, and Synergistes (Figure 1). While Thermotogae, Coprothermobacter, and Synergistes consisted of only one order each, Firmicutes and Bacteriodetes were much more diverse, containing various orders in varying compositions depending on pH conditions. Firmicutes (22–67%) and Bacteriodetes (4–15%) were more abundant in neutral and alkaline than acidic samples. Firmicutes were strongly inhibited at pH < 7.0, while the relative abundance of Bacteroidetes only decreased drastically at pH < 6.0. In contrast, Thermotogae (6–39%) were the dominant phylum at pH 5.5 and 6.0, being distinctively less abundant at pH 7.0–8.5. Coprothermobacteraeota (0–22%) and Synergistes (0–9%) were primarily present at pH 5.5. The contribution of Euryarchaeota changed little in the first three weeks of incubation, starting from approximately 6% on day 0 to 4–10% on day 21. The main increase in methanogens occurred during the next six weeks, leading to relative archaeal abundances of 4–19% on day 63. The maximum relative abundance of 16S reads and 16S gene copy number corrected reads were found in pH 6.0 and pH 7.5 samples, respectively. 

**Figure 8:**
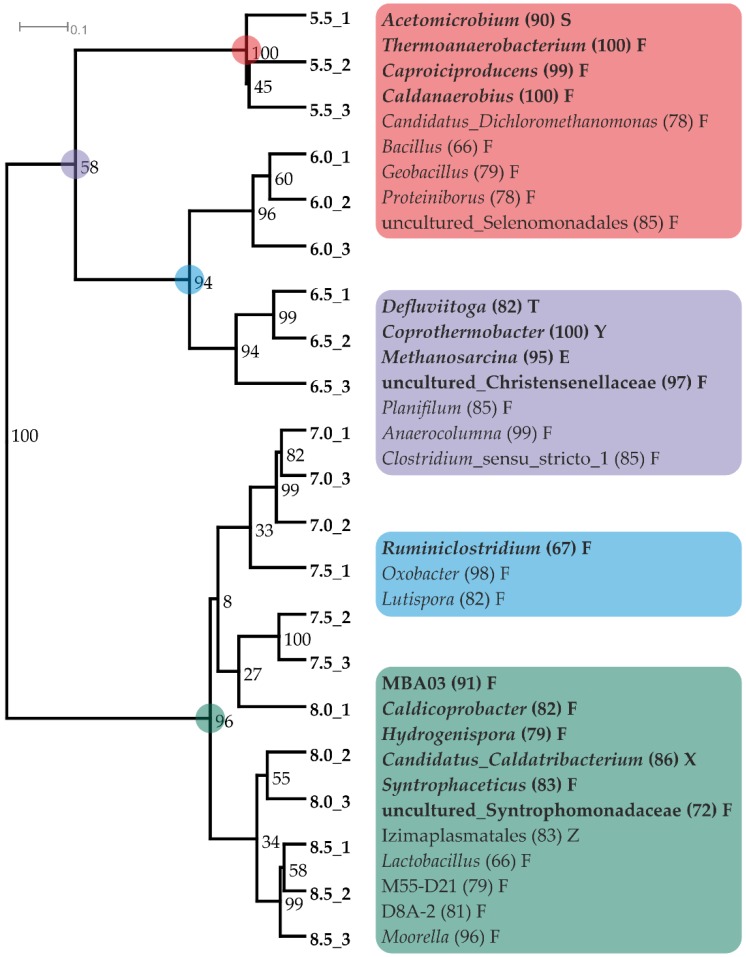
Shifts in the microbial community at varying pH values. Cluster analysis of box-cox transformed abundance data on day 63 with bootstrap numbers at nodes (Bray-Curtis, UPGMA). Colored dots link distinct subclusters with their respective indicator genera. Numbers in brackets state the IndVal (*p* < 0.01) and genera with a maximum abundance above 1% are written in bold letters. Single letters state the phyla the indicator genera; S: Synergistes, F: Firmicutes, T: Thermotogae, Y: Coprothermobacteraeota, E: Euryarchaeota, X: Atribacteria, Z: Tenericutes.

NGS data at genus level from day 21 and 63 were used for separate cluster analyses of the microbial community composition that led to almost identical results regarding both days (Figure 8, Figure S1). On day 63, two main clusters were found separating the variants with pH 7–8.5 from those with pH 5.5–6.5, with the latter comprising two sub-clusters containing only pH 5.5 and pH 6.0–6.5, respectively (Figure 8). For each of these four clusters indicator organisms could be identified. The microbial communities at pH 7.0–8.5 were mainly characterized by the genera MBA03, *Caldicoprobacter*, *Hydrogenispora*, *Candidatus Caldatribacterium*, *Syntrophaceticus*, and uncultured Syntrophomonadaceae. The more acidic samples (5.5–6.5) shared the indicator genera *Defluviitoga*, *Methanosarcina*, *Coprothermobacter* and uncultured Christensenellaceae. The genus *Ruminiclostridium* was characteristic for pH 6.0 and 6.5 samples, while *Thermoanaerobacterium*, *Acetomicrobium*, *Caldanaerobius* and *Caproiciproducens* defined the community composition at pH 5.5. A more detailed look on the relative abundances of the methanogenic members of the communities (reads corrected by mean 16S rRNA copy numbers of the genera) revealed that there was a pH dependent succession of methanogens. From acidic to alkaline, the dominant methanogens were *Methanothermobacter*, *Methanosarcina,* and *Methanoculleus*. In this context, it should be mentioned that the high 16S gene copy number of *Methanosarcina* compared to the other genera contributes strongly to the maximum of Euryarchaeota reads at pH 6.0 stated above.

## 4. Discussion

### 4.1. Impact of P-Buffer

When tested in a supplementary experiment, phosphate concentrations of >25 mM led to severe inhibitions of CH_4_ production in the initial phase of the anaerobic digestion (Figure 3). However, the CH_4_ deficits could be partially compensated during the rest of the incubation phase. 

In the main experiment, shifts in the carbon flow as well as the archaeal and bacterial community could be observed when comparing buffered and unbuffered variants with similar pH values. The 100 mM P-buffer had an inhibitory effect on the acetoclastic genus *Methanosarcina*, one of the dominant methanogens in the inoculum (Figure 7). Therefore, there was an adaption phase in P-buffer containing samples at the beginning of the experiment, during which the hydrogenotrophic genus *Methanoculleus* established. This adaptation phase caused a delayed initiation of methanogenesis and an accumulation of VFA produced during hydrolysis and acidogenesis (Figure 4). As fermentation products were hardly removed in the first three weeks, a temporary inhibition of hydrolysis and acidogenesis occurred, as can be seen in the reduced amount of reduced carbon on day 28. High VFA concentrations, altered methanogenic partners, and the presence of phosphate per se were possible causes for shifts in the bacterial community. On phylum level, the presence of P-buffer led to a shift in the relative abundance from Bacteroidetes to Firmicutes until the end of the incubation, suggesting that Firmicutes were less sensitive to the direct or indirect effect of the phosphate addition. 

The inhibitory effect of phosphate on (mostly acetoclastic) CH_4_ production was already observed by researchers investigating rice roots (50 mM, pH 7.0) and mesophilic (48 mM, pH 7.0) or thermophilic (70 mM, pH 7.1) anaerobic digestions [41,42,43]. Contrary to that, Gonzalez-Gil et al. (2002) [44] used 100 mM P-buffer (pH 6.8–7.2) in their mesophilic batch digestion fed with either acetate or a VFA mixture and did not report inhibitory effects. Further, Smith and Mah (1978) [10] grew *Methanosarcina* strain 227 successfully, in a medium containing 50 mM P-buffer at pH 6.5. 

The mode of action of the negative effect of phosphate on the AMA could not be identified conclusively. Possible mechanisms include product inhibition of the alkaline phosphatase, interferences with the metal metabolism and pH changes due to the elevated phosphate concentration [11]. Notably, the inhibition of *Methanosarcina* species did not occur in pH 6.0 samples in the present study, suggesting that the phosphate inhibition was pH dependent. In this context, the solubility of phosphate salts decreases with increasing pH, affecting the trace element availability in the system. As an example, phosphate ions precipitate magnesium together with ammonium cations to form magnesium ammonium phosphate crystals, known as struvite, within a pH range from 7.0 to 11.5 [45]. A shortage in trace elements due to phosphate precipitation might explain why the inhibitory effect of phosphate on AMA was more pronounced at neutral and alkaline pH values. 

Enzymes involved in methanogenesis are highly dependent on trace elements, e.g., cobalt, molybdenum, nickel, selenium, magnesium, iron and tungsten, as comprehensively listed in [46], with the type and amount of metals required varying with the methanogenic pathway. Investigations on the effect of trace element supplementation or limitation on methanogenic systems are common but mostly lack data on the microbial community [47]. However, Neubeck et al. (2016) [48] found that *Methanosarcina barkeri* reacted more sensitively to suboptimal nickel concentrations than the HMA *Methanobacterium bryantii* and *Methanoculleus bourgensis* when grown in pure culture. Further, Wintsche et al. (2016, 2018) [46,49] investigated the effect of trace element (cobalt, molybdenum, nickel, tungsten, iron) deprivation on the microbial community of a mesophilic lab-scale fermenter dominated by *Methanosarcina* and *Methanoculleus* species. They showed that *Methanoculleus* spp. were less affected by trace element limitations and the surviving *Methanosarcina* spp. performed increasingly hydrogenotrophic methanogenesis (HM) instead of acetoclastic methanogenesis (AM). In contrast, they observed only minor changes in the bacterial composition [49], a fact that they attributed to the higher metabolic and therefore enzymatic versatility of the bacterial community compared to the methanogenic one [49]. However, propionate oxidation by syntrophic bacteria was also shown to be sensitive to trace element shortages [50,51]. This could explain why propionate persisted in all buffered variants until the end of incubation, while it was degraded in the unbuffered control. In conclusion, trace metal limitation due to phosphate precipitation might explain the inhibiting effect of the applied P-buffer in the present study, a fact which should be considered in future investigations applying phosphate buffers.

### 4.2. Effect of pH on Ecosystem Function and Microbial Diversity

During the first three weeks, hydrolysis and acidogenesis dominated over methanogenesis, leading to the accumulation of butyrate, propionate and acetate in all samples (Figure 5). Subsequently, acid production slowed down and CH_4_ production accelerated, reducing the amount of accumulated acids. Suboptimal pH conditions affected all stages of the anaerobic digestion, reducing the amount of biogas produced and changing the composition of the involved microbial community as well as the composition of the VFA pool. Inhibitory effects of free ammonia at high pH values can be excluded, as the sludge from the biogas fermenter in Roppen was adapted to high ammonia concentrations (~350 mmol/L total ammonia (NH_4_^+^ + NH_3_); = ~30 mmol/L NH_3_ in the undiluted sludge with pH 7.5), which were above the maximum concentration of free ammonia in this study.

Previous studies on thermophilic communities from anaerobic digesters showed a large diversity in the dominant methanogenic genus. Archaeal genera commonly detected in 16S sequencing studies of thermophilic digester sludges with neutral or slightly alkaline pH were *Methanosarcina*, *Methanoculleus*, *Methanobacterium*, and *Methanothermobacter*, with *Methanosarcina* being the only acetoclastic and *Methanothermobacter* the only obligate thermophilic genus [6,52,53,54,55,56,57,58]. Concerning bacterial phyla, thermophilic communities were generally dominated by either Firmicutes or Thermotogae, and most of them also comprised, in lower abundance, Bacteroidetes and Synergistes [6,52,53,54,55,56,57,58]. The general microbial community composition in the present study was in accordance with these findings, with Firmicutes, Thermotogae, Bacteroidetes, Coprothermobacteraeota and Synergistes being the most abundant bacterial phyla and *Methanosarcina*, *Methanoculleus,* and *Methanothermobacter* being the dominant archaeal genera. The phylum Coprothermobacteraeota was created only recently [59], meaning that its members were included in the phylum Firmicutes in older publications. In contrast, the maximum abundance of the genera was not necessarily in agreement with the pH optima reported previously for cultivated members (Table 1). However, the altered pH may have allowed less competitive but more pH tolerant organisms to maintain metabolic activity at the limits of their pH range. The diversity of the microbial communities declined over time in all samples, probably as they adapted to changes in the pH and the constrained substrate spectrum with carboxymethyl cellulose as the main substrate during the batch incubation (Figure 2b). Suboptimal pH variants showed the lowest diversity, indicating that most species in the original inoculum were adapted to neutral pH values. It is commonly assumed that in most ecosystems, a high diversity indicates a high functional stability [60]. Strong perturbations of the system may, however, lead to an at least temporary reduction of the diversity, as the number of organisms adapted to atypical conditions is normally low under usual conditions [61]. In this context, it should be mentioned that thermophilic biogas microbiomes are generally less diverse than mesophilic ones due to the selective pressure of the high temperature [8]. According to cluster analysis, samples with a pH of 7.0–8.5, 6.0–6.5 and 5.5 exhibited similar community compositions within their group, meaning that pH changes between 5.5 and 6.0 and between 6.5 and 7.0 led to particularly drastic shifts in the microbial community (Figure 8). 

Despite these differences in the community composition between the single pH variants, the function of the microbiomes was largely identical, but with varying mineralization success. For each of the four major steps of anaerobic digestion, dominant genera changed with the pH but performed the same physiological tasks. To link the biochemical data characterizing the carbon flow with the 16S rRNA amplicon sequencing data, we estimated the metabolic roles of genera, which were outstandingly abundant over all samples or indicators for a specific pH, based on species descriptions of known representatives (Table 1). Although the organisms present in this investigation may differ from the described species and the species descriptions available may not cover their complete physiological potential, it will give an idea which genera shared the same niche at different pH values. Table 1 lists the maximal relative abundance of selected genera and the pH at which this maximum occurred as well as the pH range for which the respective genus acted as indicator. These results are compared with data on the pH range and the main metabolic products from available species descriptions. Therefore, abundant genera with no cultivated members are not included in Table 1.

### 4.3. Effect of pH on Physiochemical and Microbiological Aspects of the Anaerobic Digestion, Part 1: From Cellulose to Acetate 

Hydrolysis and acidogenesis were significantly pH dependent, occurring mainly in the first third of the incubation, with an optimum at pH 7.0–7.5 (Figure 5). Butyrate, propionate, and acetate accumulated in all pH variants, albeit in varying extends. In pH 5.0–6.0 samples, methanogenesis as well as hydrolysis/acidogenesis were malfunctioning and butyrate and H_2_ both reached maximal concentrations (Table S1). In this context, the extracellular pH strongly influences the toxicity of VFA, as they are only able to permeate the cell membrane in their undissociated (uncharged) form [5,22]. For instance, according to the Henderson–Hasselbalch equation at 50 °C, 16.2% and 0.19% of acetic acid are undissociated at pH 5.5 and 7.5, respectively [101]. The toxic effect is mainly caused by the intracellular dissociation of the VFA, reducing the pH of the cytoplasm and impairing the membrane proton gradient [22]. To preserve homoeostasis, microorganisms have to actively export the acids, which is increasingly energy demanding with decreasing pH [102]. In this context, Rodriguez et al. (2006) [102] and Hoelzle et al. (2014) [5] suggested that the enhanced butyrate production at acidic conditions is due to the fact that the fermentation of glucose to butyrate instead of acetate produces less acidic molecules per substrate molecule, that have to be transported outside the cell. This corresponds well to the fact that the highest butyrate concentrations were found at pH 5.0 in the present investigation.

Cellulolytic, proteolytic, and acidogenic genera with cultivated representatives, which were indicator taxa or showed high relative abundances in this study, are listed in Table 1. Abundant genera with no cultivated member included Clostridia group MBA03 and *Candidatus Caldatribacterium*, indicators for pH 7–8.5. Among the genera with cultivated members, the genus *Coprothermobacter* will be exemplarily discussed in the text, as it reached the highest abundances of all proteolytic genera, with up to 22% in pH 5.5 samples on day 63 and its role during the anaerobic digestion of cellulose is well studied. The genus was already detected in a range of thermophilic anaerobic digesters and was shown to be a potent H_2_ producer, often syntrophically associated with the HMA *Methanothermobacter* [103]. Further, a metaproteomics study of Lü et al. (2013) [104] indicated that the genus *Coprothermobacter* could be involved in syntrophic acetate oxidation (SAO). *Coprothermobacter* and *Methanothermobacter* also co-occurred in the present study, indicating that *Coprothermobacter* was an important H_2_ supplier at acidic pH values. The high abundance of the proteolytic genus in a cellulose fed system was surprising but might be caused by increased amounts of dead cells of pH intolerant microorganisms at lower pH values [71,105]. Other possible sources of proteinaceous material include extracellular enzymes, e.g., cellulases, and dead organic material from the inoculum [104]. 

The accumulated butyrate and propionate were at least partially further degraded to acetate, CO_2_ and H_2_ in all pH variants. The responsible genera could be poorly identified, as most species were not tested for butyrate or propionate oxidation during their description. Solely the OTU uncultured Syntrophomonadaceae might have included syntrophic acetate producers, as members of the genus *Syntrophomonas* are able to break down fatty acids to acetate, propionate, and H_2_ when grown syntrophically with HMA [93]. 

### 4.4. Effect of pH on Physiochemical and Microbiological Aspects of the Anaerobic Digestion, Part 2: From Acetate to CH_4_

The final cumulative CH_4_ yield, the CH_4_ production dynamics, and the dominating methanogenic pathway were strongly dependent on the prevailing pH during the incubation period (Figure 5). In samples, in which methanogenesis was not strongly inhibited (pH > 6.0), methanogenesis was bi-phasic, with most CH_4_ being produced during the second production phase lasting from day 21 to 63. The maximum cumulative CH_4_ production until day 63 was found at pH 7.5. Consistent with gas production dynamics, the second phase was also characterized by an increase in the relative abundance of Euryarchaeota, with *Methanoculleus* being the most abundant methanogenic genus at pH > 6.0. *Methanoculleus* is a HMA, metabolizing H_2_ and CO_2_ to CH_4_, which is known to be inhibited by pH < 6 [99,100]. In contrast to CO_2_, H_2_ concentrations were extremely low in pH > 6.0 samples, meaning that it was the limiting substrate for HM. During the first three weeks, H_2_ was provided by acidogenesis, which was accompanied by the accumulation of VFA, primarily acetate. However, when acidogenesis slowed down, HMA became dependent on the syntrophic oxidation of VFA for H_2_ supply. This shift in H_2_ source may explain the bi-phasic CH_4_ production in samples with a pH between 7.0 and 8.5, which contained solely hydrogenotrophic methanogens.

In contrast to butyrate and propionate, acetate was fully depleted in all samples with a pH ≥ 6.0 until the end of the incubation. While in samples with pH 6.5, *Methanoculleus* and *Methanosarcina* coexisted in similar abundances, the AMA *Methanosarcina* was not present in samples with a pH of 7.0–8.5, meaning that acetate was degraded via SAO in these samples. The known SAOB that could be detected were *Syntrophaceticus* and *Tepidanerobacter*, both indicators for the pH 7.0–8.5 cluster and members of the order Thermoanaerobacterales [93,106]. Although the only cultivated member of the genus *Syntrophaceticus* (*S. schinkii*) is mesophilic, 16S rRNA genes of related species have been detected in various thermophilic sludge communities [93,107]. The thermotolerant species *Tepidanerobacter acetatoxidans* was isolated from a mesophilic methanogenic system and is able to perform both acidogenesis and SAO [106]. Considering that the syntrophic degradation of acetate was the dominant process during the last six weeks of incubation in pH 7.0–8.5, the abundance of these two OTUs (in sum maximal 2.4%) was surprisingly low. This indicates that a range of SAOB might not have been identified as such yet, especially as most anaerobic species were not co-cultivated with H_2_ consuming partners during their characterization.

Interestingly, pH 6.0 samples were dominated by the AMA *Methanosarcina*, which seemed to be not inhibited by the P-buffer at this pH. In these samples the highest CH_4_ production rates were found in the first phase, accompanied by the lowest acetate levels after 21 days. Therefore, we can conclude that the present *Methanosarcina* members were able to quickly degrade the newly produced acetate to CH_4_, keeping acetate concentrations low. Members of the genus *Methanosarcina* are common in thermophilic anaerobic digesters with high acetate concentrations, as they have high growth rates compared to other methanogens [95,108,109,110].

Samples with an even lower pH of 5.0 or 5.5 produced small amounts of CH_4_ exclusively in the first production phase and were dominated by HMA, with *Methanothermobacter* being the most abundant methanogenic genus. The relative abundance of the genus increased in the pH 5.5 samples during the incubation, indicating that its members could tolerate the acidic conditions. Members of the genus *Methanothermobacter* were described to grow at pH 5–9 and found to be syntrophic partners of *Coprothermobacter* spp. as mentioned above [98,103]. As the accumulated acetate was not degraded in pH 5.0 and 5.5 samples although *Methanothermobacter* species were present, it can be concluded that both AM and SOA were inhibited by the low pH. This assumption is supported by the fact that relative abundances of *Methanosarcina*, *Syntrophaceticus*, and *Tepidanerobacter* were extremely low in these samples. Therefore, it can be assumed that the absence of acetate degraders, rather than the absence of methanogens, led to the cessation of CH_4_ production after the first three weeks.

To summarize, the main methanogenic pathway shifted from SAO + HM at pH 5.0 and 5.5 to AM at pH 6.0–6.5 and then again to SAO + HM at pH 6.5–8.5. The competition between SAOB and AMA for acetate was influenced by a range of environmental factors. Both pathways (regarded from acetate to CH_4_) yield the same amount of energy, but in the case of SAO + HM this energy has to support two organisms in contrast to AM, involving only one organism [111]. This fact allows AMA to grow faster and thus be more competitive for acetate than SAOB [111]. However, AMA were found to be more sensitive towards several inhibitory conditions, such as very high ammonia levels, allowing SAOB and HMA to take over [112]. In this study it could be shown that very low pH levels as well as high phosphate concentrations might also induce a shift from AM to SAO + HM.

## 5. Conclusions

Methane production at phosphate levels above 25 mM required the adaptation of the microbial community, which led to at least temporarily reduced methane yields. In 100 mM P-buffered samples, CH_4_ production showed an optimum at pH 7.5 and was strongly inhibited at pH 5.0–5.5. Low pH values inhibited further hydrolysis and acidogenesis. The results indicate that acetoclastic *Methanosarcina* species were generally able to compete with the cooperation of SAOB and HMA. However, they were easily inhibited by pH values lower than six or the presence of high phosphate concentrations, allowing HMA to dominate in these variants. The bacterial community was influenced by the pH conditions as well as by the physiology and abundance of the available methanogenic partners. Bacterial genera which were promoted by more acidic conditions included *Defluviitoga*, *Coprothermobacter*, *Acetomicrobium*, and *Thermoanerobacterium*, while the abundant but uncultured genera MB03 and DTU014, belonging to the Firmicutes, were restricted to neutral or slightly alkaline conditions. Despite these changes, ecosystem functions stayed stable over a wide pH range from 6.0 to 8.5.

## Figures and Tables

**Table 1:** Comparison of pH optima of abundant/important microbial genera in our study with physiological data on cultivated members from the literature. SAO: sytrophic acetate oxidation; AM: acetoclastic methanogenesis; HM: hydrogenotrophic methanogenesis; max. rel. abund: maximum relative abundance (16S reads not corrected for copy numbers); unc.: uncultured; Fo: formate; Ac: acetate; Pr: propionate; Bu: butyrate; Ca: caproate; La: lactate; Ma: malate; EtOH: ethanol.

Main Metabolic Task	OTU at Genus Level	This Study (data from day 63)		Literature		Source
		Max. Rel. Abund. (respective pH)	Indicator for pH	pH Range *	Main Products **	
Hydrolysis of	*Acetomicrobium*	9% (5.5)	5.5	5–9	Ac, H_2_	[62,63,64]
cellulose and	*Ruminiclostridium*	2% (6–7)	6.0–6.5	5–8	Ac, Pr, Bu, La, EtOH, H_2_	[65]
acidogenesis	*Defluviitoga*	39% (5.5)	5.5–6.5	7–8	Ac, H_2_	[66]
	*Herbinix*	4% (7)	-	7–10	Ac, Pr, Bu, EtOH, H_2_	[67,68]
Hydrolysis of	*Tepidimicrobium*	1% (5.5; 8.5)	-	6–10	Ac, Pr	[69]
proteins and	*Coprothermobacter*	22% (5.5)	5.5–6.5	4–9	Ac, Pr, H_2_	[70,71]
acidogenesis	*Proteiniphilum*	6% (8.5)	-	6–10	Ac, Pr	[72]
Acidogenesis	*Thermoanaerobacterium*	8% (5.5)	5.5	3–8	Ac, La, EtOH, H_2_	[73,74,75,76]
	*Caproicipruducens*	2% (5.5)	5.5	6–8	Ac, Bu, Ca, EtOH, H_2_	[77]
	*Caldanerobius*	1% (5.5)	5.5	4–8	Fo, Ac, La, EtOH	[78,79]
	unc. Christensenellaceae	3% (6.5)	-	6–9	Ac, Bu	[80]
	*Caldicoprobacter*	12% (7.5)	7.0–8.5	5–9	Ac, La, EtOH, H_2_	[81,82,83,84]
	*Hydrogenispora*	5% (8.5)	7.0–8.5	5–8	Ac, EtOH, H_2_	[85]
	Lentimicrobiaceae	9% (8.5)	-	7–9	Fo, Ac, Pr, Ma, H_2_	[86]
Oxidation of VFA	unc. Syntrophomonadaceae	1% (8.5)	7.0–8.5	6–9	Ac, Pr, H_2_	[87,88,89,90,91,92]
SAO	*Syntrophaceticus*	2% (7; 8.5)	7.0–8.5	6–8	Fo, H_2_	[93,94]
AM	*Methanosarcina*	16% (6)	5.5–6.5	5–9	CH_4_	[95,96,97]
HM	*Methanothermobacter*	4% (5.5)	-	5–9	CH_4_	[9,98]
	*Methanoculleus*	8% (7.5)	-	6–9	CH_4_	[99,100]

* Minimum and maximum pH, at which any cultivated member of the genus may grow. ** Carbon dioxide is produced by all listed acid or H_2_ producing genera and is therefore not included in the table. The list of H_2_ producers may be incomplete, as gaseous fermentation products were not analyzed during all species descriptions.

## Supplementary Materials

The following are available online at https://www.mdpi.com/2076-2607/8/2/286/s1, Table S1: Biochemical data during the anaerobic digestion, Figure S1: Shifts in microbial community and indicator genera at day 21, Figure S2: Composition of the microbial communities in day 21, Figure S3: Carbon flow between different pools during 63 days of anaerobic digestion.
