# Supplementary material for: pH and Phosphate Induced Shifts in Carbon Flow and Microbial Community during Thermophilic Anaerobic Digestion
Source: Microorganisms. 2020 Feb 20;8(2):286. doi: 10.3390/microorganisms8020286 (PMC7074938; doi:10.3390/microorganisms8020286)
Supplement: Supplementary file 1 [file microorganisms-08-00286-s001.zip › Suppl.docx]

Supplementary to:

pH and Phosphate Induced Shifts in Carbon Flow and Microbial Community during Thermophilic Anaerobic Digestion

Nina Lackner ^1^*, Andreas O. Wagner ^1^, Rudolf Markt ^1^ and Paul Illmer ^1,^

^1^ Department of Microbiology, Universität Innsbruck, 6020 Innsbruck, Austria; nina.lackner@uibk.ac.at, andreas.wagner@uibk.ac.at, rudolf.markt@uibk.ac.at, paul.illmer@uibk.ac.at

***** Correspondence: nina.lackner@uibk.ac.at

**Table S1.** Biochemical data describing carbon flow and biogas production during the anaerobic digestion (Means ± standard deviations of triplicates, daily maxima are bold). Significances were determined using Welch-ANOVA (* significant differences between pH variants (p<0.01); ◊ significant differences between unbuffered and pH 7.5 samples (p<0.01)). Comparisons including variants with no variation (zero values in all three parallels) gave no results. Sum of reduced carbons comprises cumulative CH_4_, acetate, propionate and butyrate.

| **Cumulative H_2_ [mmol/L medium]** | | |  |  |  |  |  |  |
| --- | --- | --- | --- | --- | --- | --- | --- | --- |
| initial pH | day 0 | day 3 | day 7 | day 14 | day 21 * | day 28 | day 42 * | day 63 |
| 5.0 | 0.00 ± 0.00 | 0.05 ± 0.01 | **0.20 ± 0.09** | **0.15 ± 0.06** | **0.10 ± 0.03** | **0.07 ± 0.02** | **0.05 ± 0.00** | **0.04 ± 0.01** |
| 5.5 | 0.00 ± 0.00 | **0.09 ± 0.05** | 0.08 ± 0.04 | 0.07 ± 0.04 | 0.06 ± 0.01 | 0.05 ± 0.01 | 0.04 ± 0.01 | 0.03 ± 0.01 |
| 6.0 | 0.00 ± 0.00 | 0.04 ± 0.01 | 0.03 ± 0.01 | 0.03 ± 0.00 | 0.02 ± 0.00 | 0.02 ± 0.01 | 0.02 ± 0.00 | 0.02 ± 0.02 |
| 6.5 | 0.00 ± 0.00 | 0.03 ± 0.00 | 0.02 ± 0.00 | 0.01 ± 0.00 | 0.01 ± 0.00 | 0.02 ± 0.01 | 0.00 ± 0.00 | 0.00 ± 0.00 |
| 7.0 | 0.00 ± 0.00 | 0.02 ± 0.01 | 0.02 ± 0.00 | 0.01 ± 0.01 | 0.01 ± 0.01 | 0.02 ± 0.00 | 0.00 ± 0.00 | 0.01 ± 0.01 |
| 7.5 | 0.00 ± 0.00 | 0.03 ± 0.00 | 0.03 ± 0.00 | 0.02 ± 0.00 | 0.02 ± 0.00 | 0.02 ± 0.00 | 0.00 ± 0.00 | 0.01 ± 0.01 |
| 8.0 | 0.00 ± 0.00 | 0.06 ± 0.02 | 0.03 ± 0.00 | 0.02 ± 0.00 | 0.02 ± 0.01 | 0.01 ± 0.01 | 0.01 ± 0.01 | 0.02 ± 0.01 |
| 8.5 | 0.00 ± 0.00 | 0.07 ± 0.03 | 0.04 ± 0.01 | 0.01 ± 0.01 | 0.03 ± 0.00 | 0.02 ± 0.00 | 0.01 ± 0.01 | 0.01 ± 0.01 |
| unbuffered | 0.00 ± 0.00 | 0.04 ± 0.01 | 0.02 ± 0.02 | 0.01 ± 0.01 | 0.01 ± 0.01 | 0.01 ± 0.00 | 0.01 ± 0.00 | 0.01 ± 0.00 |
| **Cumulative CH_4_ [mmol/L medium]** | | |  |  |  |  |  |  |
| initial pH | day 0 * | day 3 * ◊ | day 7 * ◊ | day 14 * ◊ | day 21 * ◊ | day 28 * ◊ | day 42 * | day 63 * |
| 5.0 | 0.00 ± 0.00 | 0.26 ± 0.02 | 0.82 ± 0.12 | 1.40 ± 0.04 | 1.63 ± 0.09 | 1.76 ± 0.14 | 2.16 ± 0.11 | 2.28 ± 0.13 |
| 5.5 | 0.00 ± 0.00 | 0.57 ± 0.05 | 1.56 ± 0.10 | 2.76 ± 0.53 | 3.57 ± 1.10 | 4.18 ± 1.59 | 5.16 ± 2.46 | 6.79 ± 4.42 |
| 6.0 | 0.00 ± 0.00 | 1.17 ± 0.33 | 3.63 ± 1.47 | 5.95 ± 2.53 | 8.30 ± 4.16 | 11.04 ± 4.30 | 16.83 ± 1.63 | 19.60 ± 0.45 |
| 6.5 | 0.01 ± 0.00 | 0.98 ± 0.03 | 2.63 ± 0.25 | 4.97 ± 0.26 | 6.63 ± 0.61 | 9.16 ± 1.49 | 19.94 ± 2.19 | 24.14 ± 0.68 |
| 7.0 | 0.02 ± 0.00 | **1.24 ± 0.08** | **3.30 ± 0.18** | **5.48 ± 0.11** | **8.72 ± 0.36** | **15.81 ± 0.50** | 25.13 ± 0.68 | 28.27 ± 0.24 |
| 7.5 | **0.03 ± 0.00** | 1.00 ± 0.13 | 2.78 ± 0.28 | 5.20 ± 0.28 | 7.75 ± 0.79 | 13.96 ± 1.34 | **28.23 ± 2.33** | **36.43 ± 7.00** |
| 8.0 | 0.01 ± 0.00 | 0.73 ± 0.06 | 2.40 ± 0.23 | 4.74 ± 0.48 | 6.73 ± 0.53 | 10.99 ± 1.20 | 24.20 ± 3.08 | 28.60 ± 5.01 |
| 8.5 | 0.00 ± 0.00 | 0.67 ± 0.18 | 1.91 ± 0.20 | 4.06 ± 0.22 | 5.78 ± 0.24 | 9.00 ± 0.46 | 21.44 ± 0.75 | 24.87 ± 0.95 |
| unbuffered | 0.03 ± 0.00 | 2.75 ± 0.38 | 14.31 ± 0.66 | 27.17 ± 1.30 | 33.77 ± 1.91 | 38.72 ± 1.93 | 44.11 ± 5.35 | 48.93 ± 9.67 |
| **Cumulative CO_2_ [mmol/L medium]** | | |  |  |  |  |  |  |
| initial pH | day 0 * | day 3 * | day 7 * | day 14 * ◊ | day 21 * ◊ | day 28 * ◊ | day 42 * ◊ | day 63 * ◊ |
| 5.0 | 1.29 ± 0.17 | 2.94 ± 0.25 | 4.29 ± 0.36 | 4.91 ± 0.52 | 5.19 ± 0.43 | 5.52 ± 0.63 | 5.96 ± 0.13 | 5.93 ± 0.29 |
| 5.5 | 1.14 ± 0.24 | 3.40 ± 0.48 | 4.64 ± 0.62 | 5.63 ± 0.91 | 6.35 ± 1.26 | 7.13 ± 1.78 | 7.94 ± 2.60 | 9.33 ± 4.34 |
| 6.0 | 1.40 ± 0.35 | 5.43 ± 0.62 | 7.45 ± 0.83 | 9.05 ± 1.27 | 10.87 ± 2.39 | **14.20 ± 2.41** | 17.86 ± 0.45 | 20.58 ± 0.67 |
| 6.5 | **1.86 ± 0.09** | **6.30 ± 0.15** | **8.13 ± 0.30** | **9.62 ± 0.53** | **11.18 ± 0.83** | 14.05 ± 1.39 | **18.44 ± 1.41** | **22.15 ± 1.37** |
| 7.0 | 1.56 ± 0.35 | 4.84 ± 0.36 | 6.50 ± 0.46 | 7.86 ± 0.33 | 9.18 ± 0.87 | 12.56 ± 0.62 | 15.62 ± 1.18 | 17.89 ± 0.48 |
| 7.5 | 0.72 ± 0.05 | 2.81 ± 0.22 | 4.04 ± 0.25 | 5.33 ± 0.23 | 6.29 ± 0.23 | 7.81 ± 0.62 | 12.36 ± 1.73 | 17.41 ± 4.44 |
| 8.0 | 0.40 ± 0.15 | 1.56 ± 0.13 | 2.41 ± 0.22 | 3.52 ± 0.26 | 4.27 ± 0.32 | 5.28 ± 0.49 | 7.47 ± 1.19 | 9.90 ± 2.62 |
| 8.5 | 0.49 ± 0.43 | 1.03 ± 0.05 | 1.57 ± 0.10 | 2.42 ± 0.15 | 3.05 ± 0.30 | 3.67 ± 0.25 | 5.00 ± 0.45 | 6.64 ± 0.45 |
| unbuffered | 0.96 ± 0.07 | 4.50 ± 0.68 | 9.38 ± 2.06 | 17.13 ± 1.87 | 22.15 ± 2.41 | 26.30 ± 2.32 | 31.92 ± 1.60 | 37.67 ± 1.38 |
| **Acetate [mmolC/L medium]** | |  |  |  |  |  |  |  |
| initial pH | day 0 * | day 3 * | day 7 * ◊ | day 14 * ◊ | day 21 * ◊ | day 28 * ◊ | day 42 | day 63 * |
| 5.0 | 6.41 ± 0.23 | 5.03 ± 0.22 | 7.23 ± 0.44 | 9.84 ± 0.28 | 11.92 ± 0.25 | 12.46 ± 0.08 | **11.17 ± 0.57** | **14.31 ± 0.17** |
| 5.5 | 6.54 ± 0.28 | 4.62 ± 0.66 | 6.73 ± 1.38 | 9.28 ± 1.09 | 11.11 ± 1.50 | 11.50 ± 1.99 | 9.03 ± 2.68 | 9.72 ± 6.78 |
| 6.0 | 6.88 ± 0.57 | 5.75 ± 0.61 | 6.85 ± 1.43 | 7.85 ± 3.24 | 7.40 ± 6.33 | 5.67 ± 5.98 | 0.70 ± 1.21 | 0.05 ± 0.09 |
| 6.5 | 7.39 ± 0.28 | 7.55 ± 0.34 | 11.11 ± 0.05 | 15.18 ± 0.25 | 17.45 ± 0.28 | 15.82 ± 1.73 | 2.61 ± 1.76 | 0.33 ± 0.04 |
| 7.0 | 7.02 ± 0.47 | 8.67 ± 0.33 | 13.95 ± 0.29 | 17.31 ± 0.15 | 18.58 ± 0.18 | 10.07 ± 0.34 | 0.19 ± 0.33 | 0.40 ± 0.11 |
| 7.5 | **7.84 ± 0.21** | 9.73 ± 0.96 | **15.69 ± 1.17** | **17.46 ± 0.91** | 18.97 ± 0.59 | 12.01 ± 1.35 | 0.17 ± 0.30 | 0.38 ± 0.14 |
| 8.0 | 7.83 ± 0.13 | 8.09 ± 1.09 | 14.53 ± 0.85 | 16.51 ± 1.15 | **19.61 ± 1.01** | **16.01 ± 0.88** | 0.00 ± 0.00 | 0.26 ± 0.03 |
| 8.5 | 7.82 ± 0.40 | **10.13 ± 1.14** | 13.36 ± 1.22 | 15.33 ± 1.08 | 18.77 ± 0.94 | 15.86 ± 1.04 | 0.18 ± 0.31 | 0.18 ± 0.16 |
| unbuffered | 8.04 ± 0.35 | 7.67 ± 0.70 | 1.71 ± 0.15 | 0.09 ± 0.15 | 0.17 ± 0.06 | 0.03 ± 0.05 | 0.00 ± 0.00 | 0.00 ± 0.00 |
| **Propionate [mmolC/L medium]** | | |  |  |  |  |  |  |
| initial pH | day 0 * | day 3 * | day 7 * | day 14 * | day 21 * ◊ | day 28 * ◊ | day 42 * | day 63 * |
| 5.0 | 1.24 ± 0.12 | 2.50 ± 0.12 | 1.08 ± 0.22 | 3.48 ± 0.12 | 1.88 ± 0.23 | 1.78 ± 0.11 | 3.47 ± 0.06 | 2.27 ± 0.09 |
| 5.5 | 0.93 ± 0.27 | 2.24 ± 0.09 | 1.08 ± 0.27 | 4.54 ± 0.29 | 1.96 ± 0.03 | 1.89 ± 0.03 | 3.95 ± 0.05 | 2.61 ± 0.11 |
| 6.0 | 1.41 ± 0.23 | 2.07 ± 0.32 | 1.01 ± 0.11 | 4.89 ± 0.29 | 1.92 ± 0.29 | 2.03 ± 0.33 | 4.66 ± 0.35 | 3.01 ± 0.26 |
| 6.5 | 1.64 ± 0.26 | 2.23 ± 0.12 | 1.48 ± 0.14 | **5.03 ± 0.07** | 2.57 ± 0.10 | 2.70 ± 0.05 | 6.63 ± 0.32 | 4.57 ± 0.05 |
| 7.0 | 1.54 ± 0.11 | 2.79 ± 0.45 | 1.63 ± 0.14 | 4.81 ± 0.20 | 2.69 ± 0.11 | 2.89 ± 0.11 | 7.55 ± 0.40 | 4.95 ± 0.15 |
| 7.5 | 2.02 ± 0.39 | **3.37 ± 0.30** | 1.98 ± 0.15 | 4.59 ± 0.30 | **2.89 ± 0.30** | **3.04 ± 0.30** | **8.44 ± 0.12** | **6.41 ± 1.02** |
| 8.0 | 1.87 ± 0.38 | 0.96 ± 0.27 | 1.90 ± 0.25 | 3.98 ± 0.30 | 2.49 ± 0.18 | 2.90 ± 0.20 | 6.53 ± 0.51 | 4.53 ± 0.66 |
| 8.5 | **2.23 ± 0.12** | 1.83 ± 0.20 | **2.13 ± 0.24** | 2.71 ± 1.53 | 2.27 ± 0.23 | 2.59 ± 0.22 | 6.26 ± 0.34 | 4.47 ± 0.34 |
| unbuffered | 1.79 ± 0.33 | 2.29 ± 0.32 | 3.58 ± 0.48 | 0.00 ± 0.00 | 0.56 ± 0.48 | 0.86 ± 0.06 | 0.00 ± 0.00 | 0.00 ± 0.00 |

**Table S1ff.** Biochemical data describing carbon flow and biogas production during the anaerobic digestion (Means ± standard deviations of triplicates, daily maxima of pH variants are bold). Significances were determined using Welch-ANOVA (* significant differences between pH variants (p<0.01); ◊ significant differences between unbuffered and pH 7.5 samples (p<0.01)). Comparisons including variants with no variation (zero values in all three parallels) gave no results. Sum of reduced carbons comprises cumulative CH_4_, acetate, propionate and butyrate.

| **Butyrate [mmolC/L medium]** | |  |  |  |  |  |  |  |
| --- | --- | --- | --- | --- | --- | --- | --- | --- |
| initial pH | day 0 | day 3 | day 7 | day 14 * | day 21 * | day 28 | day 42 | day 63 * |
| 5.0 | 0.00 ± 0.00 | 0.00 ± 0.00 | 0.23 ± 0.21 | **1.55 ± 0.17** | **2.47 ± 0.34** | **2.88 ± 0.22** | **1.73 ± 0.15** | **3.35 ± 0.22** |
| 5.5 | 0.00 ± 0.00 | 0.00 ± 0.00 | 0.24 ± 0.26 | 1.13 ± 0.08 | 1.92 ± 0.08 | 2.29 ± 0.09 | 0.96 ± 0.45 | 2.61 ± 0.10 |
| 6.0 | 0.00 ± 0.00 | 0.00 ± 0.00 | 0.20 ± 0.04 | 0.35 ± 0.08 | 1.55 ± 0.06 | 1.84 ± 0.08 | 0.21 ± 0.37 | 1.28 ± 0.73 |
| 6.5 | 0.00 ± 0.00 | 0.00 ± 0.00 | 0.36 ± 0.04 | 0.43 ± 0.06 | 1.59 ± 0.02 | 1.96 ± 0.04 | 0.61 ± 0.17 | 0.92 ± 0.00 |
| 7.0 | 0.00 ± 0.00 | 0.00 ± 0.00 | 0.23 ± 0.06 | 0.45 ± 0.02 | 1.43 ± 0.02 | 1.64 ± 0.00 | 0.00 ± 0.00 | 0.00 ± 0.00 |
| 7.5 | 0.00 ± 0.00 | 0.51 ± 0.45 | **0.39 ± 0.12** | 0.51 ± 0.06 | 1.45 ± 0.02 | 1.13 ± 0.50 | 0.00 ± 0.00 | 0.00 ± 0.00 |
| 8.0 | 0.00 ± 0.00 | **0.77 ± 0.15** | 0.29 ± 0.05 | 0.88 ± 0.31 | 1.56 ± 0.11 | 1.45 ± 0.14 | 0.48 ± 0.04 | 0.84 ± 0.04 |
| 8.5 | 0.00 ± 0.00 | 0.60 ± 0.31 | 0.37 ± 0.05 | 0.81 ± 0.41 | 1.68 ± 0.14 | 1.77 ± 0.15 | 0.53 ± 0.06 | 0.92 ± 0.08 |
| unbuffered | 0.40 ± 0.38 | 0.25 ± 0.06 | 0.00 ± 0.00 | 0.00 ± 0.00 | 0.00 ± 0.00 | 0.00 ± 0.00 | 0.00 ± 0.00 | 0.00 ± 0.00 |
| **Sum of reduced carbon [mmolC/L medium]** | | |  |  |  |  |  |  |
| initial pH | day 0 * | day 3 * | day 7 * | day 14 * | day 21 * | day 28 * ◊ | day 42 * | day 63 * |
| 5.0 | 7.66 ± 0.11 | 7.79 ± 0.27 | 9.36 ± 0.96 | 16.26 ± 0.49 | 17.90 ± 0.72 | 18.88 ± 0.19 | 18.53 ± 0.47 | 22.21 ± 0.32 |
| 5.5 | 7.47 ± 0.50 | 7.43 ± 0.74 | 9.61 ± 2.00 | 17.71 ± 0.76 | 18.56 ± 0.46 | 19.87 ± 0.55 | 19.11 ± 0.38 | 21.74 ± 2.33 |
| 6.0 | 8.29 ± 0.39 | 8.98 ± 0.70 | 11.69 ± 0.28 | 19.04 ± 0.81 | 19.17 ± 1.98 | 20.57 ± 1.37 | 22.41 ± 0.99 | 23.95 ± 0.20 |
| 6.5 | 9.04 ± 0.37 | 10.75 ± 0.41 | 15.58 ± 0.11 | 25.61 ± 0.54 | 28.24 ± 0.42 | 29.64 ± 0.35 | 29.79 ± 0.87 | 29.95 ± 0.75 |
| 7.0 | 8.58 ± 0.36 | 12.70 ± 0.69 | 19.11 ± 0.52 | **28.05 ± 0.20** | **31.41 ± 0.11** | 30.41 ± 0.18 | 32.88 ± 0.73 | 33.62 ± 0.07 |
| 7.5 | 9.89 ± 0.60 | **14.61 ± 0.88** | **20.84 ± 1.44** | 27.75 ± 1.53 | 31.07 ± 1.68 | 30.14 ± 0.59 | **36.84 ± 2.60** | **43.22 ± 8.08** |
| 8.0 | 9.72 ± 0.24 | 10.55 ± 1.19 | 19.12 ± 1.04 | 26.11 ± 1.80 | 30.39 ± 1.69 | **31.35 ± 1.66** | 31.21 ± 3.61 | 34.23 ± 5.66 |
| 8.5 | **10.05 ± 0.34** | 13.24 ± 1.63 | 17.77 ± 1.69 | 22.91 ± 2.97 | 28.49 ± 1.38 | 29.22 ± 1.20 | 28.41 ± 0.98 | 30.44 ± 1.28 |
| unbuffered | 10.26 ± 1.04 | 12.97 ± 0.77 | 19.60 ± 0.33 | 27.26 ± 1.42 | 34.50 ± 1.82 | 39.60 ± 1.96 | 44.11 ± 5.35 | 48.93 ± 9.67 |


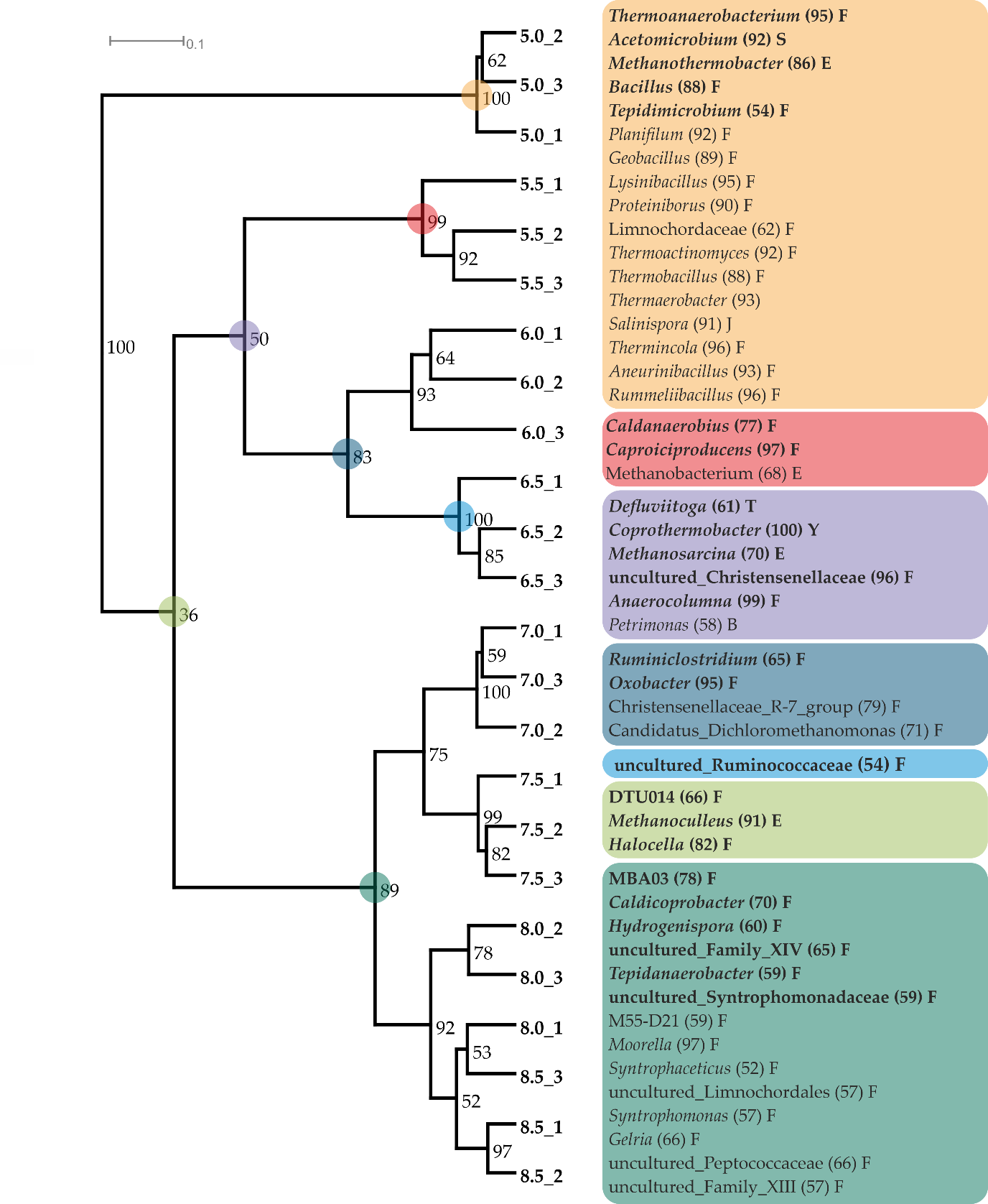


**Figure S1.** Shifts in the microbial community at varying pH values. Cluster analysis of box-cox transformed abundance data on day 21 with bootstrap numbers at nodes (Bray-Curtis, UPGMA). Colored dots link distinct subclusters with their respective indicator genera. Numbers in brackets state the IndVal (p<0.01) and genera showing maximum abundances above 1% are written in bold letters. Single letters state the phyla the indicator genera; F: Firmicutes, S: Synergistes, J: Actinobacteria, E: Euryarchaeota, T: Thermotogae, Y: Coprothermobacter, B: Bacteriodetes.


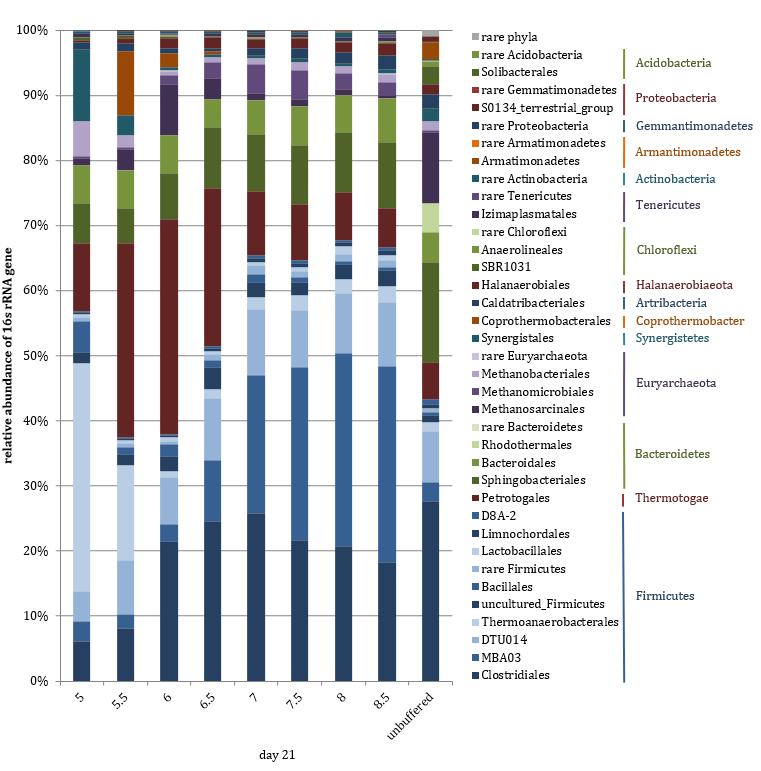


**Figure S2**. Microbial community composition at phylum and order level of samples incubated for 21 days at varying pH conditions (100 mM P-buffered) or unbuffered. Phyla or orders with less than 1% abundance in any sample were summarized as rare phyla or rare members of the particular phylum, respectively (means of triplicates).


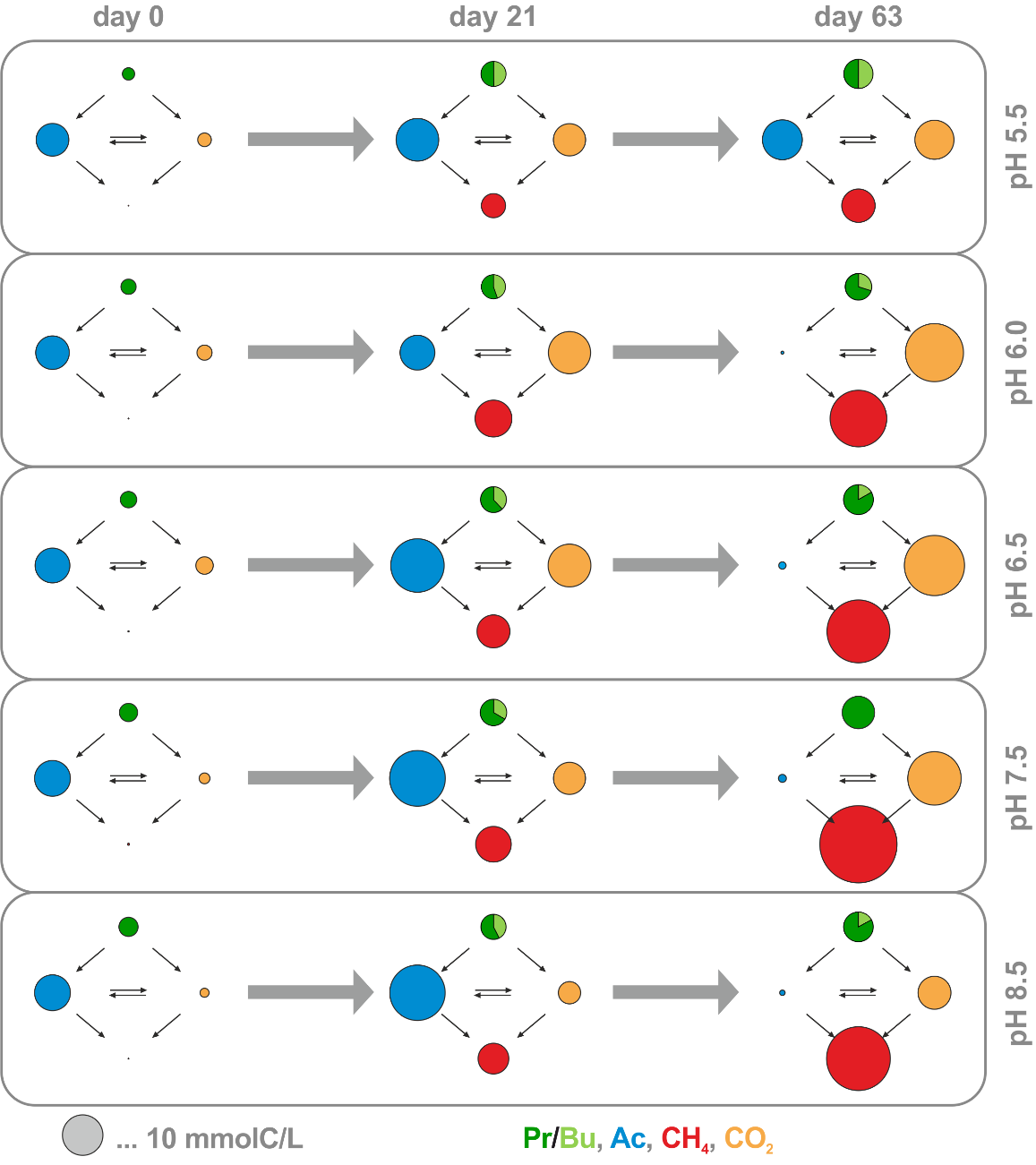


**Figure S3:** Carbon flow between different pools during 63 days of anaerobic digestion at varying pH conditions. Pr/Bu: sum of carbon in propionate and butyrate, Ac: carbon in acetate. Areas of the colored circles correspond to mmolC/L medium.
